# Modulation of Gi Proteins in Hypertension: Role of Angiotensin II and Oxidative Stress

**DOI:** 10.2174/157340310793566046

**Published:** 2010-11

**Authors:** Madhu B Anand-Srivastava

**Affiliations:** Department of Physiology, Faculty of Medicine, University of Montreal, Québec, Canada

**Keywords:** G-proteins, Angiotensin II, oxidative stress, MAPkinase, adenylyl cyclase, hypertension.

## Abstract

Guanine nucleotide regulatory proteins (G-proteins) play a key role in the regulation of various signal transduction systems including adenylyl cyclase/cAMP and phospholipase C (PLC)/phosphatidyl inositol turnover (PI). These are implicated in the modulation of a variety of physiological functions such as platelet functions, cardiovascular functions, including arterial tone and reactivity. Several abnormalities in adenylyl cyclase activity, cAMP levels and G proteins have shown to be responsible for the altered cardiac performance and vascular functions observed in cardiovascular disease states. The enhanced or unaltered levels of inhibitory G-proteins (Giα-2 and Giα-3) and mRNA have been reported in different models of hypertension, whereas Gsα levels were shown to be unaltered. These changes in G-protein expression were associated with Gi functions. The enhanced levels of Giα proteins precede the development of blood pressure and suggest that overexpression of Gi proteins may be one of the contributing factors for the pathogenesis of hypertension. The augmented levels of vasoactive peptides, including angiotensin II (AngII), were shown to contribute to enhanced expression of Giα proteins and associated adenylyl cyclase signaling and thereby increased blood pressure. In addition, enhanced oxidative stress in hypertension due to Ang II may also be responsible for the enhanced expression of Giα proteins observed in hypertension. The mechanism by which oxidative stress enhances the expression of Gi proteins appears to be through the activation of mitogen activated protein (MAP) kinase activity.

## INTRODUCTION

Guanine nucleotide regulatory proteins (G-proteins) are a family of guanosine triphosphate (GTP) binding proteins that play a key regulatory role as transducers in a variety of signal transduction system. These include adenylyl cyclase/cAMP system [1], the receptor-mediated activation of phospholipase C and A2 [2,3] and a number of hormone-and neurotransmitter-regulated ionic channels [4,5]. G proteins are heterotrimeric proteins composed of three distinct subunits; α, β and γ [6]. The α-subunits bind and hydrolyse GTP and confer specificity in receptor and effector interactions [6]. The GDP bound form of α binds tightly to βγ and is inactive, whereas the GTP bound form of α dissociates from βγ and serves as a regulator of effector proteins. All α-subunits possess intrinsic GTPase activity and hydrolyse the terminal phosphate of bound GTP to yield bound GDP and free inorganic phosphate (Pi). Upon hormone binding and receptor activation, the receptor interacts with the heterotrimeric protein to promote a conformational change and dissociation of bound GDP from the guanine nucleotide binding site. GDP is released and replaced by GTP. Binding of GTP to α induces a conformational change and promotes the dissociation of hormone receptor complex (HR) and the holo G protein into α and βγ. Both α-GTP and βγ subunits can interact with effectors. This activation cycle is terminated by intrinsic GTPase activity of α-subunit. The GDP-bound form of α- subunit has high affinity for βγ andthen reassociates with the βγ dimmer to form the hetero trimer in the basal resting state. The family of G-protein α-subunits can be subclassified according to functional or structural relationship. More than 20 mammalian Gα gene products and several alternatively spliced isoforms have been identified. These can be divided into four major sub-families according to amino acid homology and are represented by Gsα, Giα, Gqα/α11 and α12/α13. The G proteins Gsα and Giα are implicated in the regulation of adenylyl cyclase/cAMP signal transduction system.

The hormone-sensitive adenylyl cyclase system is composed of three components: the receptor, the catalytic subunit, and G-proteins – stimulatory (Gs) and inhibitory (Gi). Molecular cloning has revealed four different forms of Gsα having molecular weights of 45, 45, 52 kD resulting from the different splicing of one gene [7-9]. Gsα is positively coupled to adenylyl cyclase and mediates the stimulatory responses of hormones on adenylyl cyclase [10,11]. The Gs-mediated activation of adenylyl cyclase results in the increase formation of cAMP. cAMP activates cAMP-dependent protein kinase A that induces the phosphorylation of contractile filaments, sarcolemmal and sarcoplasmic proteins, and regulates intracellular calcium homeostasis [12]. In addition, Gsα was also shown to open the Ca^2+^ channels directly by a cAMP-independent mechanism [13]. In contrast, Giα protein is associated with adenylyl cyclase inhibition [10,11]. Three distinct forms of Giα, namely, Giα_-1_, Giα_-2,_ and Giα_-3_ have been cloned and encoded by three distinct genes [14-16]. All three forms of Giα (Giα_1-3_) have been shown to be implicated in adenylyl cyclase inhibition [17] and activation of atrial Ach-K^+^ channels [18]. Both the Gα and Gβγ dimer mediate G-protein signaling. Five different β subunits of 35-36 kDa and 12 γ subunits of 8-10 kDa have been identified by molecular cloning. The βγ dimer is tightly associated with GDP bound chain and facilitate the interaction of G-protein with a receptor molecule. The effectors regulated by Gβγ include K^+^ channels, phospholipase C-β, and adenylyl cyclase [19-21].

G-protein α-subunits also possess specific residues that can be covalently modified by bacterial toxins. Cholera toxin catalyzes the transfer of ADP-ribose moiety of NAD to a specific arginine residue in certain α-subunits, whereas pertussis toxin ADP-ribosylates those α-subunits that contain a specific cysteine residue near to the carboxy terminus. Modification of α-subunit by cholera toxin persistently activates these protein by inhibiting their GTPase activity, whereas pertussis toxin inactives Giα protein and thereby results in the uncoupling of receptor from the effector.

## G-PROTEINS AND MEMBRANE SIGNALING IN CARDIOVASCULAR DISEASE

A number of cardiovascular disease states that eventually result in chronic congestive heart failure are associated with alterations in cardiac performance. Several hormonal factors such as angiotensin II, endothelin and alterations in signal transduction mechanisms including adenylyl cyclase and phospholipase C (PLC) have been reported to play an important role in the alterations of cardiac performance [22].

## G-PROTEINS AND MEMBRANE SIGNALING IN HYPERTENSION

Alterations in G-protein levels and functions such as altered adenylyl cyclase responsiveness to various agonists have also been demonstrated in cardiovascular and non cardiovascular tissues from genetic as well as experimental hypertensive rats [23-28].

An overexpression of Giα_-2_ and Giα_-3_ proteins as well as  their genes was shown in hearts and aorta from spontaneously hypertensive rats (SHRs), deoxycorticosterone acetate (DOCA)-salt hypertensive rats (HR), N-[Omega]-nitro-L-arginine methylester, (L-NAME) HR and 1 kidney 1 clip (1K1C) HR [23-33], whereas Gsα protein and its gene was not altered in SHRs, 1K1C and L-NAME HR, and was decreased in DOCA-salt HRs [23-25,28,31-33].  In addition, the levels of Goα in hearts from SHR were unaltered [24]. Alterations in Gi-protein levels have been shown to be reflected in altered responsiveness of adenylyl cyclase to stimulatory and inhibitory hormones in SHRs, and experimental models of hypertensive rats [24,26,27,31-33].  However, a decreased expression of Giα proteins was also shown in different tissues from different model of HR including Milan hypertensive rats (MHS) [34-36].  The VSMC from MHS exhibit enhanced basal adenylyl cyclase activity as compared to control normotensive rats (MNS).  The number of β-adrenoceptors and the stimulations exerted by isoproterenol and prostaglandin E1 (PGE_1_) were significantly increased in MHS than in MNS.  On the other hand, platelets from SHRs [26] as well as from hypertensive patients [37] exhibited a decreased expression of Gi_α-2_ and Gi_α-3_ protein as compared to Wistar-Kyoto (WKY) and to normotensive control subjects, respectively whereas the levels of Gs_α_ protein were not altered. The decreased expression of Gi_α-2_ and Gi_α-3_ was correlated with adenylyl cyclase inhibition by inhibitory hormones.  The ANP and Ang II-mediated inhibitions were completely attenuated in platelets from SHRs and hypertensive patients, whereas the stimulatory effects of PGE_1_, NECA and forskolin were augmented [26,37].  However, McLellan *et* *al*. [38] were unable to show any changes in the levels of Gsα, Giα-2 and Gβ in platelets from hypertensive patients as compared to normotensive subjects, whereas an enhanced stimulation of adenylyl cyclase by PGE_1_ was observed in hypertensive patients as compared to normotensive subjects. On the other hand, lymphocytes from SHRs [39] and hypertensive patients [40] showed a decreased responsiveness of adenylyl cyclase to stimulatory hormones, which may be attributed to the alterations in Gs and Gi proteins.  The potentiation of stimulatory responses of several hormones on adenylyl cyclase has also been demonstrated in platelets and splenocytic membranes from SHRs [41,42].  In addition, antihypertensive drug therapy (a combination of β-blockers Ca^2+^ channel blocker, ACE inhibitor, etc.) partially restored Giα-_2_ levels and the enhanced stimulations exerted by hormones toward normotensive subjects [37]. These effects on platelet function may underlie the beneficial effects of antihypertensive agents on some of the complications of hypertension.  

In addition, the levels of Gsα, Giα_1-3,_ Goα, and Gβ were also shown to be unaltered in myocardium from SHRs, whereas hormonal stimulations of adenylyl cyclase were reduced in SHRs, and FSK-stimulated enzyme activity was greater in SHRs as compared to WKY [43]. The reduction in the hormone receptor binding sites may be one of the possible mechanisms responsible for such an impaired response of hormones [44-46]. However, the decreased stimulation of adenylyl cyclase by dopamine D-1 receptors in the kidney tubules from SHRs was shown to be attributed to the defective coupling and not to the changes in the receptor number [47].

Furthermore, the increased levels of Giα were shown to be associated with hypertension and not with hypertrophy, due to the fact that heart and aorta from Nω-nitro-L-arginine methyl ester-(L-NAME)-induced hypertensive rats, which do not have cardiac hypertrophy exhibited enhanced levels of Giα_-2_ and Giα_-3 _proteins as well as mRNA, whereas the levels of Gsα protein were unaltered [31]. The increased levels of Giα_-2 _and Giα_-3_ proteins and their mRNA in heart and aorta precedes the development of blood pressure in SHRs [48], and in DOCA-salt hypertensive rats [49], and suggest that the enhanced levels of Giα proteins which result in the decreased levels of cAMP may be one of the contributing factors in the pathogenesis of hypertension. This was further supported by the recent studies showing that the inactivation of Giα protein in prehypertensive rats (2 week old SHR) by a single injection of pertussis toxin (PT) (1.5 μg/100 gm body weight) prevented the development of high blood pressure which was associated with PT-induced decreased levels of Giα proteins [50]. Furthermore, Triggle *et al*. [51] have also shown that treatment of the SHRs (adult) with PT lowered blood pressure.

## ROLE OF ENDOGENOUS ANGIOTENSIN II IN ENHANCED EXPRESSION OF GI PROTEINS IN HYPERTENSION

**Fig. (1):**
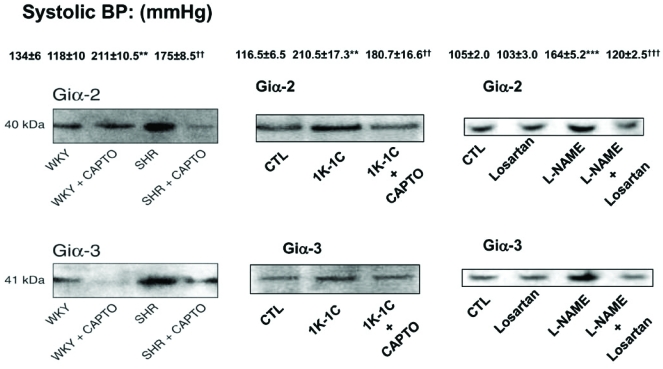
Effect of Captopril and losartan treatment on blood pressure and the expression of Gi proteins in different models of hypertensive rats (HR). 12 week-old SHR and age-matched WKY rats, 1 kidney 1 clip hypertensive rats (1K1C HR) were treated with captopril (150mg/kgB.W/day) as described earlier [32,61] whereas L-NAME-induced hypertensive rats were treated with losartan (10mg/kg B.W/day) as described earlier [62]. The blood pressure was monitored by the tail cuff method. The expression of Giα-2 and Giα-3 protein in heart from SHR and L-NAME HR and aorta from 1K1C HR were determinated by Western blotting using antibodies AS/7 and EC/1 against Giα-2 and Giα-3 protein respectively. The blots are representative of 4 separate experiments. ^**^P<0.01, ^***^P<0.001 vs control/WKY, ^††^P<0.01, ^†††^P<0.001 vs SHR/1K1C/L-NAME.

The levels of vasoactive peptides such as angiotensin II (Ang II), endothelin (ET-1) and arginine vasopressin (AVP), as well as growth factors that have been reported to be augmented in various models of hypertension [52-60], may be responsible for the enhanced expression of Giα proteins in hypertension. In this regard, a role of Ang II in enhanced expression of Giα protein in SHR and 1K1C hypertensive rats (HR) has been suggested by the studies showing that captopril; an angiotensin coverting enzyme (ACE) inhibitor treatment of the SHR and 1K1C HR that decreased the blood pressure also restored the enhanced levels of Giα protein to control levels (Fig. **1**). Similarly, the increased blood pressure and enhanced expression of Giα proteins in L-NAME hypertensive rats was also shown to be restored to control levels by losartan, an AT1 receptor antagonist (Fig. **1**) suggesting the implication of Ang II in increased levels of Giα proteins and increased blood pressure in L-NAME-induced hypertension. These treatments were also shown to restore the diminished stimulation of adenylyl cyclase by stimulatory hormones and enhanced inhibition by inhibitory hormones observed in SHRs, 1K1C and L-NAME HR [32, 61,62]. In addition, infusion of Ang II in rats that increased blood pressure has also been reported to enhance the levels of Giα proteins [63]. Similarly, nitrendipin and fosinopril treatments have also been reported to have similar effects on Gi proteins and functions in hearts from SHRs [64] and further support the implication of Ang II in enhanced levels of Giα protein in SHR.

## ROLE OF OXIDATIVE STRESS IN ENHANCED EXPRESSION OF GI PROTEINS IN HYPERTENSION

Reactive oxygen species (ROS) such as O_2_^-^, OH^-^ and H_2_O_2_ that cause oxidative stress have been shown to play a major role in the pathophysiology of cardiovascular diseases including hypertension [65]. NADPH oxidase is the enzyme responsible for the formation of O_2_^-^ from the O_2_ molecule. Vascular NADPH oxidase is multimeric protein complex composed of at least 4 components: cell membrane-associated p^22phox^ and gp^91phox^ (or gp^91phox ^[Nox^2^] homologues, nox1 and nox4), and cytosolic subunits, p47phox and p67phox [66,67]. The levels of ROS, have been shown to be augmented in spontaneous (genetic) SHR and experimental hypertension as well as in patients with various hypertensive disorders [68-76]. In addition, the enhanced expression of different subunits of NADPH oxidase such as p^47phox^, Nox^4^, p^2phox ^that has been shown in several tissues from SHR [72, 75,76], appear to be responsible for the enhanced activity of NADPH oxidase and ROS production in SHR.

**Fig. (2):**
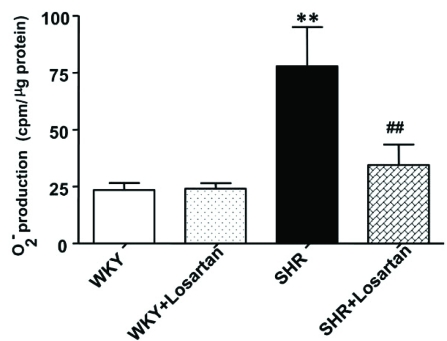
Effect of losartan on superoxide anion (O2^-^) production in vascular smooth muscle cells (VSMC) from Wistar–Kyoto (WKY) and spontaneously hypertensive rats (SHR). VSMC from 12-week-old SHR and age-matched WKY rats were treated with 10-5 mol/l losartan for 24 h, and O2^-^-production was determined as described earlier [77]. Data presented as the mean ± SEM of 5 separate experiments. ^**^P < 0.01 versus WKY rats, ^#^P < 0.05 versus SHR. Adapted from [77].

**Fig. (5):**
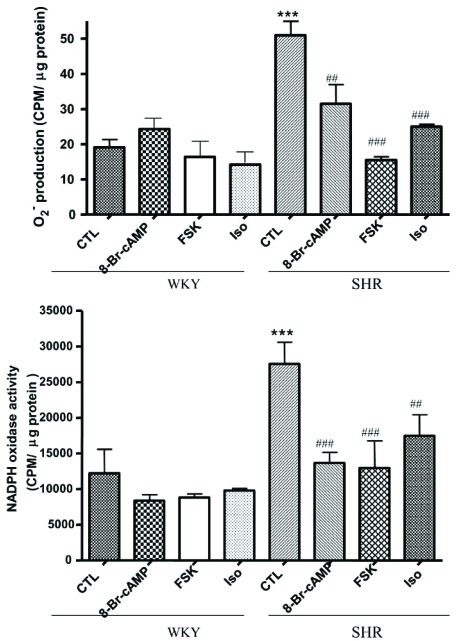
Effect of forskolin (FSK), isoproterenol (Iso) and 8Br-cAMP on superoxide anion (O_2_^-^) production in vascular smooth muscle cells (VSMC) from 12-week-old spontaneously hypertensive rat (SHR) and age matched Wistar-Kyoto (WKY) rats. VSMC from SHR and WKY rats were pretreated with FSK (100 µM), Iso (50 µM) and 8Br-cAMP (0.5 mM) for 24 h, and O_2_^-^ production (**A**) and NADPH oxidase activity (**B**) was determined as described earlier [80] Values are mean ± SEM of 3 separate experiments. ^***^p < 0.001 vs WKY, ^##^p < 0.01, ^###^p < 0.001 vs SHR. Adapted from [80].

**Fig. (6):**
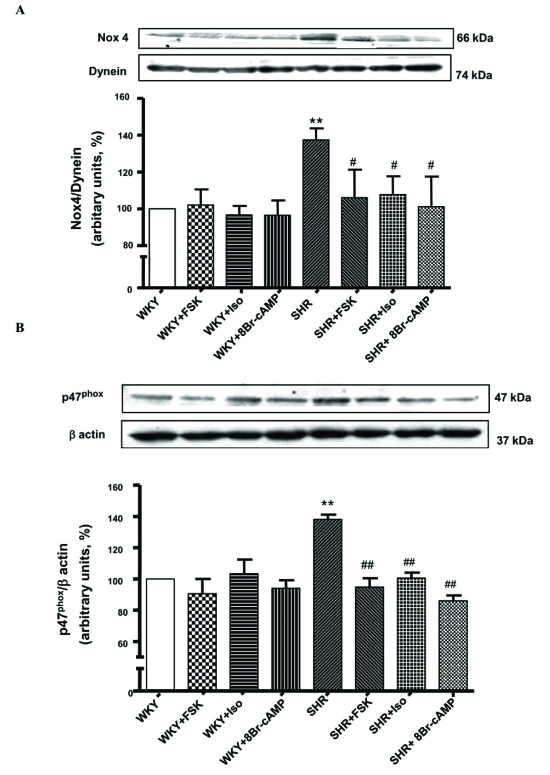
Effect of forskolin (FSK), isoproterenol (Iso) and 8Br-cAMP on the levels of Nox4 and p^47phox^ protein expression in vascular smooth muscle cells (VSMC) from 12-week-old spontaneously hypertensive rats (SHR) and age matched Wistar-Kyoto (WKY) rats. VSMC from SHR and WKY rats were pretreated without (control) or with FSK (100 µM), Iso (50 µM) and 8Br cAMP (0.5 mM) for 24 hr. Membrane proteins (30 µg) were separated and transferred to nitrocellulose, which was then immunoblotted using specific antibodies against Nox4 (N-15, A) and p^47phox^ (C-20,B) as described earlier [78] The Dynein or β actin was used to assess the loading of the protein. Immunoblots are representative of 4 separate experiments. Lower panels: the graph shows quantification of protein ratio of Nox^4^/Dynein (**A**) or p^47phox^/β actin bands by densitometric scanning. The results are expressed as percentage of WKY taken as 100%. Values are mean ± SEM of 4 separate experiments. ^**^p < 0.01 vs WKY, ^#^p < 0.05, ## < 0.01 vs SHR. Adapted from [78].

We showed recently that VSMC from SHR exhibit enhanced levels of O_2_^-^ [77] which were attenuated by AT1 receptor antagonist, losartan (Fig. **2**). In addition, the expression of p^47phox^ and Nox^4^ was also augmented in these cells as shown in Fig. (**3**) [78]. Ang II, whose levels are augmented in hypertension, has been shown to be one of the important factors regulating NADPH oxidase. In this regard treatment of A10 VSMC with AngII has been shown to augment the production of O_2_^- ^and the expression of Nox^4^ and p^47phox^ proteins of NADPH oxidase [79]. In addition, we also showed that the enhanced levels of Giα proteins in SHR may also contribute to the enhanced production of O_2_^- ^and increased NADPH oxidase activity, because pertussis toxin treatment of VSMC from SHR also attenuated the enhanced levels of O_2_^-^ and enhanced activity of NADPH oxidase to control WKY levels as shown in Fig. (**4**) [78]. Furthermore, the decreased levels of cAMP in VSMC from SHR have also been reported to contribute to the enhanced production of O_2_^- ^and increased activity of NADPH oxidase because the treatment of VSMC from SHR with 8Br-cAMP, as well as with cAMP elevating agents such as isoproterenol and forskolin (FSK), restored the enhanced activity of NADPH oxidase and enhanced levels of O_2_^-^ (Fig. **5**) and p^47phox^ and Nox^4^ to control WKY levels (Fig. **6**). In addition, a role of reduced levels of cAMP in enhanced oxidative stress was further supported by the fact that Ang II-evoked enhanced production of O_2_^-^, NADPH oxidase activity and enhanced levels of p47phox and Nox4 proteins were shown to be restored to control levels by 8Br-cAMP in A10 VSMC [80].

**Fig. (7):**
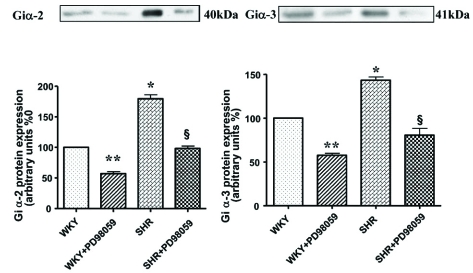
Effect of PD 98059 on Giα-2 and Giα-3 protein expression in VSMC from 12 week-old SHR and age-matched WKY rats. Confluent VSMC from SHR and WKY rats were treated with or without PD 98059 (10µM) for 24 hours at 37°C. Membrane proteins (30 *µ*g) were separated and transferred to nitrocellulose, which was then immunoblotted with specific antibodies against Giα-2 (**A**) and Giα-3 (**B**)  as described earlier [77]. The blots are representative of three separate experiments. The graphs at lower panel show quantification of protein bands by densitometric scanning. The results are expressed as percentage of WKY control which has been taken as 100%. Values are mean ± S.E.M of 5 separate experiments *P < 0.05 , ^**^P<0.01 vs WKY, ^δ^P < 0.05 vs SHR. . Adapted from [77].

**Fig. (8):**
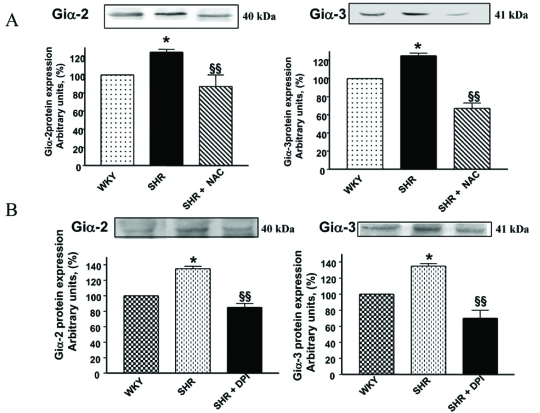
Effect of N-acetyl-L-cysteine (NAC) and diphenyleneiodonium (DPI).on Giα-2 and Giα-3 protein expression in vascular smooth muscle cells (VSMC) from 12 week-old SHR and age-matched WKY rats. Confluent VSMC from SHR and WKY rats were treated with 20 mM NAC(**A**) or 10µM DPI(**B**) for 24 hours at 37°C. Membrane proteins (30 *µ*g) were separated and transferred to nitrocellulose, which was immunoblotted with antibodies AS/7 and EC/1 against Giα-2 and Giα-3 respectively  as described earlier [77] The blots are representative of 5 separate experiments. The graphs at lower panel show quantification of protein bands by densitometric scanning. The results are expressed as percentage of WKY control which has been taken as 100%. Values are mean ± S.E.M of 5 separate experiments *P < 0.05 vs WKY, ^δδ^P < 0.01 vs SHR. Adapted from [77].

**Fig. (9):**
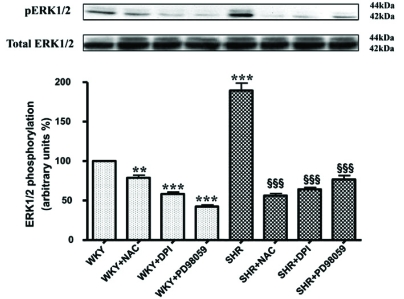
Effect of NAC and DPI on ERK 1/2 phosphorylation in vascular smooth muscle cells (VSMC) from 12 week-old SHR and WKY rats. Confluent VSMC from SHR and WKY rats were treated with 20 mM N-acetyl-L-cysteine (NAC) or 10 µM diphenyleneiodonium (DPI) for 24 hours at 37°C. Cell lysates were immunoblotted by phospho-specif-Tyr^204^-ERK1/2 antibodies as shown at the top panel. Blots were also analyzed for total ERK1/2 (bottom panel). The blots are representative of 3 separate experiments. Detection of p-ERK1/2 and total ERK1/2 was performed with chemiluminesecence Western blotting detection reagents. WKY levels were taken as 100%. Values are mean ± S.E.M of 3 separate experiments. ^**^P<0.01, ^***^P < 0.001 vs WKYand ^δδδ^P < 0.001 vs SHR. Adapted from [77].

**Fig. (10):**
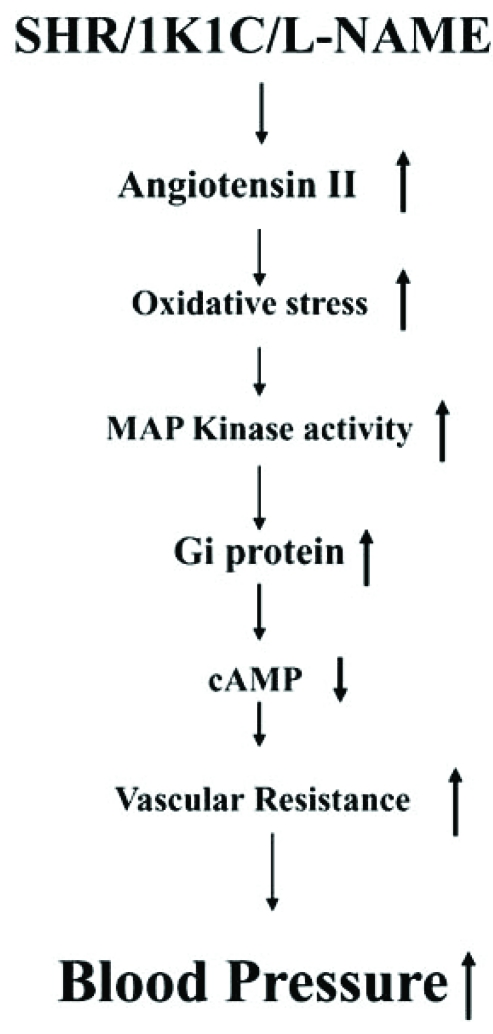
Possible mechanisms involving angiotensin II and oxidative stress in enhanced Gi protein expression in hypertension. Gi protein expression is enhanced in genetic (SHR) and experimental hypertension including 1 kidney 1 clip (1K1C) and L-NAME –induced hypertension. Inhibition of nitric oxide synthase (NOS) by L-NAME activates renin angiotensin system, and also decreases the level of NO. 1K1C hypertensive rats also exhibit enhanced levels of AngII. Ang II increases oxidative stress that through increased MAP kinase activity results in enhanced expression of Giα proteins and thereby hypertension.

The role of MAP kinase and PI3K signaling in Ang II-induced enhanced levels of Giα proteins has also been reported [79, 81]. In addition, MEK inhibitor PD98059 (Fig.**7**) as well as antioxidants such as diphenyleneiodonium (DPI) and N-acetyl cysteine (NAC) (Fig. **8**) were also shown to restore the enhanced levels of Giα proteins in SHR to control WKY levels further suggest the implication of MAP kinase and oxidative stress in the enhanced expression of Giα protein in SHR [77]. Furthermore, VSMC from SHR exhibited the enhanced phosphorylation of ERK1/2 which was also restored to WKY levels by antioxidants (Fig. **9**) [77] and suggest that enhanced oxidative stress through MAP kinase signaling may contribute to the enhanced expression of Giα protein in SHR (Fig. **10**).

## CONCLUSIONS

In conclusion, we have discussed the alterations in G-proteins and associated functions in hypertension. We have mainly focused on Gi and Gs proteins which are implicated in the regulation of the adenylyl cyclase/cAMP signal transduction system that play an important role in the regulation of cardiovascular functions, including vascular tone and reactivity and cell proliferation. The levels of Giα-2 and Giα-3 proteins and mRNA are increased in hearts and aorta from genetic and experimentally induced hypertensive rats, whereas the levels of Gsα are unaltered in genetic and decreased in experimentally induced hypertensive rats with established hypertrophy. The increased levels of Giα-2 and Giα-3 are associated with increased Gi functions, resulting in greater decreases in cAMP levels, which may partly explain the increased vascular resistance in hypertension. On the other hand, the decreased levels of Gsα and decreased formation of cAMP in hypertension associated with hypertrophy may also contribute to the increased vascular reactivity in hypertension. The increased levels of Giα-2 and Giα-3 may contribute to the pathogenesis of hypertension whereas decreased levels of Gsα may be associated with hypertrophy and not with hypertension. This notion is substantiated by our recent studies, showing that enhanced expression of Giα-2 and Giα-3 proteins and mRNA precede the development of blood pressure. The role of enhanced levels of Giα proteins in the pathogenesis of hypertension was further supported by our studies showing that inactivation of Giα proteins by pertussis toxin treatment in prehypertensive SHR prevented the development of blood pressure. However, the levels of Gs were decreased only in 15 weeks of SHRs with established hypertrophy. Similarly, L-NAME hypertensive rats that do not have cardiac hypertrophy exhibited enhanced expression of Giα-2 and Giα-3 and no changes in Gsα, whereas hypertrophied rats with volume-overload hypertrophy, which do not have hypertension, exhibited decreased levels of Gsα and no augmentation in Giα-2 or Giα-3 proteins. The increased oxidative stress due to enhanced levels of endogenous vasoactive peptides including AngII through MAP kinase signaling, may contribute to the augmented levels of Gi proteins in SHR. Thus, taken together, it can be concluded that decreased formation of cAMP levels, either by increased levels and function of Gi or decreased levels of Gsα and associated functions, may be responsible for the altered cardiac performance and vascular reactivity in cardiovascular disease including hypertension.

## Figures and Tables

**Fig. (3):**
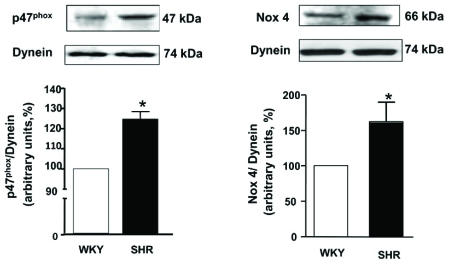
The expression of Nox4 and p47 ^phox^ protein in vascular smooth muscle cells (VSMC) from Wistar–Kyoto (WKY) and spontaneously hypertensive rats (SHR). Membrane proteins (30µg) were separated and transferred to nitrocellulose, which was then immunoblotted by using specific antibody against (**A**) p47 ^phox^ (C-20) and (**B**) Nox4 (N-15). The dynein or β-actin was used to assess the loading protein. The results are expressed as percentage of WKY rats taken as 100%. Values are means ± SE of 4 separate experiments. ^*^p<0.05 vs WKY. Adapted from [78].

**Fig. (4):**
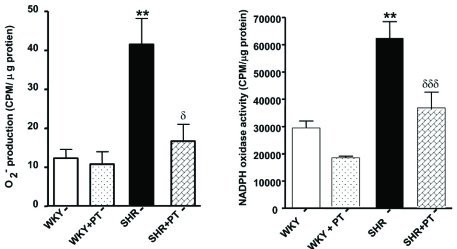
Effect of pertussis toxin treatment on superoxide anion (O_2_^-^) production (**A**) and NADPH oxidase activity (**B**) in vascular smooth muscle cells (VSMC) from 12-week-old spontaneously hypertensive rats (SHR) and age-matched Wistar-Kyoto (WKY) rats. VSMC from SHR and WKY rats were pretreated with 0.5µg/ml pertussis toxin for 24 hr as described earlier [50] and O_2_^-^ production and NADPH oxidase activity was determined as described earlier [78]. Values are mean ± SEM of 3 separate experiments. ^**^p<0.01, ^***^p < 0.001 vs WKY, ^δ^p < 0.05, ^δδ^p<0.01 vs SHR. Adapted from [78].
